# FEASIBILITY AND ACCEPTABILITY PILOT OF THE ENHANCING ACTIVE CAREGIVER TRAINING (ENACT) INTERVENTION

**DOI:** 10.1093/geroni/igae098.2467

**Published:** 2024-12-31

**Authors:** Jacqueline Eaton, Sarah Neller, Moroni Fernandez Cajavilca, Amber Dayley, Julene Johnson, Lee Ellington

**Affiliations:** University of Utah, Salt Lake City, Utah, United States; University of Tennessee Knoxville, Knoxville, Tennessee, United States; New York University, New York, New York, United States; University of Utah, Salt Lake City, Utah, United States; University of California San Francisco, San Francisco, California, United States; University of Utah, Salt Lake City, Utah, United States

## Abstract

The process of actively engaging dementia caregivers in interventions has been insufficiently studied. This limits our understanding on how best to improve interventions. Enhancing Active Caregiver Training (EnACT) was developed to facilitate active engagement using participant-informed vignettes that portray caregiving experiences. We conducted a randomized waitlist-controlled pilot study to test the feasibility and acceptability of EnACT with a group of 30 caregivers of persons living with dementia. Participants were randomly assigned to intervention (n=15) or control (n=15) groups. The intervention group received three EnACT sessions during the first 7 weeks, which was then repeated for the control. Sessions used the vignettes to facilitate small-group dialogue, exploration, and reflection. These were delivered by trained interventionists via Zoom and each session lasted 60 minutes. Feasibility and acceptability data were collected to assess 1) interventionist training and delivery, 2) acceptability of treatment conditions, 3) participant adherence, and 4) recruitment, randomization, and assessment protocols. Preliminary results suggest that EnACT is feasible and acceptable to caregivers and interventionists. Retention rates were high (97%) and twenty-nine participants (97%) completed all six outcome surveys. Levels of satisfaction improved across the intervention, with the highest satisfaction focused on interventionists, engagement, topics, length, and accessibility. Data from this pilot is being used to prepare and plan for a fully powered randomized controlled trial.

